# Occupational physicians’ perceived barriers and suggested solutions to improve adherence to a guideline on mental health problems: analysis of a peer group training

**DOI:** 10.1186/s12913-016-1530-3

**Published:** 2016-07-16

**Authors:** Marjolein Lugtenberg, Karlijn M. van Beurden, Evelien P. M. Brouwers, Berend Terluin, Jaap van Weeghel, Jac J. L. van der Klink, Margot C. W. Joosen

**Affiliations:** Tilburg University, Tilburg School of Social and Behavioral Sciences, Tranzo Scientific Center for Care and Welfare, PO Box 90153, 5000 LE Tilburg, The Netherlands; Erasmus University Medical Center Rotterdam, Department of Public Health, PO Box 2040, 3000 CA Rotterdam, The Netherlands; Department of General Practice and Elderly Care Medicine, VU University Medical Center Amsterdam, EMGO Institute for Health and Care Research, PO Box 7057, 1007 MB Amsterdam, The Netherlands; Phrenos Centre of Expertise, PO Box 1203, 3500 BE Utrecht, The Netherlands; Parnassia Group, Dijk en Duin Mental Health Center, PO Box 305, 1900 AH Castricum, The Netherlands

**Keywords:** Mental health, Practice guideline, Occupational medicine, Barriers, Solutions, Implementation

## Abstract

**Background:**

Despite the impact of mental health problems on sickness absence, only few occupational health guidelines addressing these problems are available. Moreover, adherence has found to be suboptimal. To improve adherence to the Dutch guideline on mental health problems a training was developed for Dutch occupational physicians (OPs) focusing on identifying barriers and addressing them. The aim of this study was to provide an overview of the barriers that OPs perceived in adhering to the Dutch guideline on mental health problems as well as their solutions to overcome them.

**Methods:**

A qualitative study was conducted using data from the peer group training. Thirty-two (6 groups of 4 to 6) OPs received a multiple-session interactive training over the course of a year, focusing on identifying and addressing barriers, using a Plan-Do-Check-Act approach. Sessions were audio-taped and transcribed verbatim. Thematic content analysis was performed by two researchers with a selection of 50 % (21 out of 42) of the transcripts to identify the perceived barriers and the suggested solutions, using AtlasTi 7.0.

**Results:**

Knowledge-related barriers were perceived regarding the content of all parts of the guideline. Commonly perceived attitude-related barriers were a lack of self-efficacy to perform certain guideline recommendations and difficulties with changing habits and routines. External barriers that were commonly perceived were work-contextual barriers, such as a lack of time/work pressure, tight contracts between occupational health services (OHSs) and employers, and conflicting policy of and a lack of collaboration with other parties (e.g. employer, other healthcare providers). The most often tested solutions by OPs during the training were sharing information, experiences, tips and tricks and referring to existing tools, or developing new tools to facilitate guideline usage.

**Conclusions:**

Dutch OPs perceive a range of knowledge-related, attitude-related and external barriers in adhering to the guideline on mental health problems. The tested solutions during the training particularly seemed to focus on knowledge and attitude-related barriers. To optimally implement this or similar mental health guidelines, it may be important to complement guideline training and education of individual or groups of OPs, with interventions that address external barriers such as changing tight contracts, or improving communication and collaboration with other parties.

## Background

Mental health problems, such as depression or anxiety, are among the leading causes of work disability worldwide [1, 2]. It is estimated that at any one moment 20 % of the working-age population is suffering from a mental disorder [3], which negatively impacts work capacity and productivity [3–5] and may lead to sick leave and long-lasting work disability [6]. In the Netherlands, currently more workers are sick-listed due to mental health problems as compared to physical complaints [7]. Apart from the individual burden, associated economic and societal costs are substantial [8, 9].

Despite their major impact on sickness absence and associated individual and societal consequences, only few clinical practice guidelines addressing mental health problems as they relate to occupational health are available worldwide [10]. Among these guidelines is the Dutch guideline entitled ‘The management of workers with common mental health problems by occupational physicians (OPs)’ [11], which was developed in 2000 by The Netherlands Society of Occupational Physicians (NVAB, in Dutch) and revised in 2007 [12]. One of the central aspects of this guideline is for OPs to follow an activating approach aimed at establishing earlier return to work and lower recurrence of sickness among workers.

Whereas various activities have been performed to implement the guideline among the target group of Dutch OPs, research indicates that OPs’ adherence to the guideline’s recommendations in practice is suboptimal [13–15]. OPs do, however, report a positive attitude towards the guideline in general and the intention to use it [13]. In addition, there is some evidence for a positive association between adherence to the guideline and a shortened sick leave duration for workers with adjustment disorders [14] and minor stress-related disorders [16], as well as for common mental health problems in general [15].

To improve guideline adherence, identification of the perceived barriers among the target group, is usually considered to be a first important step [17, 18]. As opposed to other healthcare settings, few barrier studies have been conducted among occupational health care professionals such as OPs [19]. It is generally recommended that barriers should be identified at different levels (e.g. the professional, the organization, the wider environment) [17, 18]. In addition, it may be useful to study barriers over time, as they may vary across different stages of implementation [20]. Results from the analysis of barriers can, subsequently, be used as input to develop tailored interventions [17, 18, 21]. Integrating the target groups’ preferences for interventions or solutions may also be useful, as acceptance by the target group is crucial for successful implementation and behavior change [22].

As part of a randomized controlled trial (RCT), which aimed to explore how the management of sick-listed workers by OPs can be improved [23], a tailored implementation strategy was developed for OPs to improve their adherence to the guideline on mental health problems. OPs received a multiple-session interactive peer group training focusing on identifying and addressing barriers [24]. The training was perceived as a feasible and much appreciated method among participating OPs [24]. The current paper aims to provide an overview of the barriers that OPs perceived in adhering to the guideline on mental health problems in practice as well as of the solutions they came up with to address them.

## Methods

### Setting

In the Netherlands, approximately 2000 OPs [25] assist employers and workers in occupational health issues, safety and sickness absence management by providing occupational health care to the working population [26]. The OP has a central role in the Dutch social security system, by providing advice to both employers and workers during the return to work process. If reported sick, Dutch workers are required to visit an OP for independent assessment and reintegration plan. Employers are obligated to hire OPs. Most Dutch employers have contracts with independently operating Organizational Health Services (OHSs). OPs employed at an OHS therefore often work for several companies at multiple locations.

Since 1998, the NVAB, has developed and implemented evidence-based practice guidelines for OPs for a variety of conditions and diseases [27]. One of these guidelines is ‘The management of workers with common mental health problems by OPs’, which was developed in 2000 [11] and revised in 2007 [12].

### Description of the guideline on mental health problems

The guideline [11, 12] recommends OPs follow an activating approach in both case and care management. The content of the guideline is based on cognitive behavioral principles aiming to enhance the problem solving capacity of workers, particularly in relation to their work context [11, 12] and is expected to result in earlier return to work and lower recurrence of sickness among (sick-listed) workers. The guideline consists of four different parts, which can be considered as consecutive steps [11, 12], as described in Table 1. Besides the core guideline document, supporting documents are available for OPs such as guideline-related tools (i.e. the rumination exercise and metaphors) [12].

**Table 1:** Summary of the guideline on mental health problems [12]

Part of the guideline	Content
1. Problem orientation and diagnosis	An early involvement of the OP in the sick leave process of the worker is promoted (first consultation about 2 weeks after the worker reports sick). A simplified classification of mental health problems is introduced in four categories: i) stress-related complaints, ii) depression, iii) anxiety disorder, and iv) other psychiatric disorders. Furthermore, problem inventory should focus on factors related to the worker, his or her work environment, and the interaction between these two.
2. Intervention/Treatment	The OP acts as case manager by monitoring and evaluating the process of recovery (process-based evaluation). When recovery stagnates, the OP should intervene by acting as care manager by using cognitive behavioral techniques to enhance the problem-solving capacity of the worker, providing the worker and work environment with information/advice on the recovery and the RTW process, contacting the GP when problems remain or increase, and referring the worker to a specialized intervention when necessary. In addition, the OP should advise the work environment (e.g., supervisors, managers, and human resource managers) on how to support the worker and enhance the recovery and RTW process.
3. Relapse prevention	The integration of relapse prevention from the first contact with the worker is achieved by enhancing the problem-solving capacity of the worker. The newly acquired problem solving skills are resumed in at least one specific relapse prevention meeting after RTW.
4. Evaluation	During follow-up meetings, evaluation of the recovery process includes the perspectives of the worker, supervisor, and other professionals involved. Follow-up meetings with the worker should take place every 3 weeks during the first 3 months, and every 6 weeks thereafter. The supervisor or work environment should be contacted once a month. Follow-up contacts with the GP or other professionals should take place when the recovery process stagnates or when there is doubt about the diagnosis or treatment.

*OPs* occupational physicians, *RTW* return-to-work, *GP* general practitioner

### Study design

A qualitative study was conducted using data from a multiple-session small interactive peer group training for Dutch OPs. The training was part of a trial focusing on the reduction of sick leave duration due to common mental disorders among workers [23].

The training was developed for OPs as a tailored implementation strategy aimed at improving adherence to the guideline on mental health problems. The training used a Plan-Do-Check-Act (PDCA) approach, which provides a method for structuring change related to quality improvement [28, 29]. Small-scale settings are usually promoted within this approach, as this enables rapid assessment and provides flexibility to adapt the change according to feedback, to ensure that fit-for-purpose solutions are developed [29]. Moreover, the multiple-session design of the training offers the opportunity to explore the evolution of perceived barriers among the target group over time. For instance, knowledge-related barriers may be most relevant at the beginning of the implementation process, whereas, once these have been removed, the existence of attitude-related and external barriers may come forward [24].

The way this tailored implementation strategy was carried out and received by OPs is described elsewhere [24]. The current paper provides an overview of the barriers that OPs perceived in adhering to the guideline on mental health problems in practice, as well as of their solutions to overcome them, as identified during the training sessions.

The RATS checklist [30], a checklist designed for reporting qualitative research, was used – whenever applicable - in this paper.

### Participant selection

To select participants for the trial all OPs that were employed at a large OHS in the southern part of the Netherlands (*N* = approx. 155) were invited to participate. First, the researchers (MJ and EB) presented their research proposal of the trial [23] at several meetings for OPs at the OHS, after which OPs could register to participate. Subsequently, an invitation was sent by email to all OPs; a reminder email was sent after two weeks. Finally, all OPs who had not yet responded were invited by telephone by one of the researchers (MJ).

A total of 66 OPs agreed to participate and, after giving written consent, were randomized to either the intervention group (*N* = 32) or the control group or (*N* = 34). All 32 OPs from the intervention group were to receive the training. The 32 OPs were divided into six groups of 4–6 OPs, based on their work locations.

### Content of the training

The training sessions were held at six regional offices of the OHS across the southern part of the Netherlands; each group attended the training at one location. The training consisted of eight two-hour-meetings which were scheduled over the course of a year.

The sessions were moderated by MJ (principal researcher of the study and experienced trainer of groups) and in 5 sessions EB (supervisor of the study) was also present. In the first meeting the trainer introduced herself, explained her role as a researcher and emphasized her independence towards the guideline. After providing basic information about the training (structure of training, role of participants, confidential setting, anonymity in reporting) the formal training started.

The training was specifically developed for this study and was aimed at improving OPs’ adherence to the guideline on mental health problems. A multiple-session training protocol was used [24]. The training followed the consecutive steps of the guideline, with each session focusing on a different topic. A PDCA approach was used to structure the discussions. In each session the perceived barriers in adhering to that specific topic were discussed. Next, OPs suggested solutions to address these barriers, taking into account the context of their daily practice. Subsequently, solutions were tested by the OPs in their daily practice. Finally, results were evaluated and, when necessary, solutions were adjusted. This PDCA cycle was repeated in each meeting and for all topics stated in the guideline. The trainer (MJ) guided the groups by structuring the meetings, facilitating the discussions and monitoring the progress [24]. All 48 sessions were audio-taped.

### Data analysis and synthesis

Forty-two out of 48 sessions were transcribed verbatim. The first training session of each group (*n* = 6) was not transcribed as this session was an introduction session and did not focus on specific barriers and interventions.

Because of the large amount of remaining material (6 groups × 7 sessions × 2 h = 84 h) and the overlap in perceived barriers and tested solutions between groups, we chose to analyze the transcripts of 3 out of 6 (50 %) groups for each session. Groups were chosen based on maximizing variation in perceived barriers and tested solutions. For each training session we started selecting the group that first attended this session, because of the possible spillover effect of the trainer with the subsequent groups of each session. The two other groups of each session were chosen based on summary reports of the training, which were assessed by the trainer in terms of adding variance in perceived barriers and solutions to address them. A total of 21 transcripts was selected.

Two researchers (ML and MJ) conducted both inductive and deductive thematic content analysis [31] using the software program Atlas.ti 7.0. ML and MJ studied the transcripts of the first group that attended a training session independently and created a code list of the identified barriers as well as of the interventions. Next, the code lists were compared and discrepancies were discussed until consensus was reached and one code list was created. Subsequently, one of the researchers (ML) coded the transcripts of the remaining two selected groups of each session, while a second coder (KvB, EB, JvW, JvdK), checked these transcripts using the agreed on code list.

To categorize the barriers we used the framework of Cabana et al. as a basis [19], and additional (sub) barriers were formulated if needed. In this framework, three main groups of barriers to follow guidelines are distinguished: knowledge-related barriers, attitude-related barriers and external barriers, which are each subdivided into several other barriers. To achieve adherence, all relevant barriers must be tackled. Whereas the framework of Cabana initially focused on guidelines as a whole, it has been recommended to analyze barriers at the level of the specific key recommendations [32, 33], as this is the concrete behavioral level. To categorize the solutions we used ‘open coding’ as the solutions were very specific and tailor-made and did not fit in existing models of interventions.

Next, the final code list was discussed by two researchers (ML and MJ) and emerging themes were grouped into a code tree and reflected upon. This process resulted in an overview of barriers to using the guideline on mental health problem as well as an overview of tested solutions.

## Results

### Description of participants

Of the 32 OPs who had agreed to participate in the training, one OP decided not to participate in the training after all, due to time constraints. The remaining 31 OPs attended all eight meetings. Six OPs were not able to attend a training session of their own group, but joined another group to attend that particular training meeting.

The mean age of the 31 participants was 53 years (SD = 4.3) and 17 (55 %) were male. On average, the OPs had 21 years (SD = 7.1) of experience working as an OP and were working 33 h a week (SD = 5.6); 28 OPs (90 %) had previously received education on the Dutch guideline through continuing medical education. Compared to the total population of Dutch OPs female OPs were slightly overrepresented [25].

### Perceived barriers

Table 2 presents an overview of the barriers that were identified among OPs to using the guideline on mental health problems. They can, following Cabana [19], be divided into three main categories: 1. Knowledge-related barriers, 2. Attitude-related barriers, and 3. External barriers.

**Table 2:** Overview of perceived barriers to using the guideline on mental health problems among OPs^a^

1. Knowledge-related barriers
- Lack of knowledge
Lack of knowledge of (content of) guideline recommendations
Lack of knowledge of availability of guideline-related tools (e.g. rumination exercise, metaphors)
2. Attitude-related barriers
- Lack of agreement guidelines in general
Lack of agreement with the concept of guidelines (e.g. perceiving them as too dogmatic, involving too much bureaucracy, too rigid to apply, not practical).
- Lack of agreement with this specific guideline
Lack of agreement with the guideline due to a lack of applicability of its recommendations in practice (e.g. perceiving practice as more complex than guideline and not being able to capture reality in the guideline).
- Lack of self-efficacy
Lack of believe that one can actually perform a behavior or guideline recommendation.
- Lack of outcome expectancy
Lack of believe that a given behavior will actually lead to a particular consequence.
- Inertia of previous practice
Experiencing difficulties with changing habits and routines in order to learn new things.
3. External barriers
- Worker factors
Perceiving worker factors as difficult in adhering to the guideline (e.g. worker preferences, demands, behavior).
- Guideline factors
Perceiving the guideline or its recommendations as difficult in adhering to the guideline (e.g. not clear, verbose, inconsistent, too complex of a terminology, not easy to read/readable).
- Work-contextual factors
Perceiving factors in the work-context of the OP as difficult in adhering to the guideline, such as:
→ Work pressure/Lack of time
→ Setting OPs operate in (e.g. difficult setting in terms of the role OPs have in assessments, questioning their independency towards the worker)
→ Organizational constraints
○ Policy of OHS (e.g. policy with respect to work pressure)
○ Non-user friendly computer systems (e.g. difficult to use/conflicting with one another)
○ Lack of resources/practical constraints (e.g. not having tools available when working at several locations)
→ Contracts between OHSs and employers (e.g. too tight arrangements in terms of available time/reimbursement)
→ Conflicting policy of and lack of collaboration with other parties
○ Employer policy (e.g. conflicting policy with respect to what is best for workers in terms of working/not working, the provided care, non-work-related problems)
○ Collaboration with employer (e.g. no adequate arrangements in terms of roles and treatment).
○ Policy of other disciplines (GP, psychologists etc.) (e.g. conflicting policy with respect to type and course of treatment, taking factor work into account)
○ Collaboration with other disciplines (GP, psychologists etc.) (e.g. no adequate arrangements in terms of communication, reporting and feedback)
→ Fear of misuse of information/control by others (e.g. fear that medical practice data will be used for other purposes by disciplinary jurisdiction or by Dutch Institute for Employee Benefit Schemes) (UWV in Dutch) etc.)

^a^For which the framework of barriers of Cabana et al. [19] was used as a basis to classify the perceived barriers to guideline adherence

*OP(s)* occupational physician(s), *OHS(s)* occupational health service(s), *GP* general practitioner, *UWV* Dutch Institute for Employee Benefit Schemes (UWV in Dutch)

#### Knowledge-related barriers

OPs reported a lack of knowledge regarding the content of all four parts of the guideline and the availability of guideline-related tools (Table 2). For example, some OPs were not familiar with one of the central aspects of the guideline, i.e. that a stronger emphasis is put on evaluating the process of recovery (a process-based evaluation) rather than on working time contingent. Others were not aware of the availability of guideline-related tools, such as the rumination exercise (i.e. an exercise to help control rumination or worrying). In addition, whereas some OPs believed they knew the guideline quite well, they discovered during the training that they did not really understand it after all.

#### Attitude-related barriers

Several attitude-related barriers were reported (Table 2). *Lack of agreement with guidelines in general* was mentioned as a barrier among OPs. Some OPs indicated that they felt that guidelines involve too much bureaucracy, and can therefore be rather cumbersome. They indicated to needing latitude not to work exclusively according to models and schemes. Also, a perceived *lack of agreement with this specific guideline* due to a lack of applicability was mentioned to be a barrier:

*“I notice that I – I do want to apply the guideline, but not as strictly as it’s formulated…I mean: you just cannot catch real-life cases in this single guideline. It’s always different or more complicated or harder …”.*

Another commonly perceived attitude-related barrier was *lack of self-efficacy*, i.e. not feeling capable of performing certain guideline recommendations due to a perceived lack of training or experience. OPs indicated, for example, that they did not know how to educate or provide information to the working environment of the workers and that they lacked tools to assist them in this:

*“I am still not sure how to explain supervisors in like half an hour….I want to guide them and show them a better way to handle the situation”.*

*Lack of outcome expectancy* was reported as a barrier by OPs. Some OPs indicated that, for example, they did not believe structural relapse prevention really makes a difference as they assumed that the organization will not take any further actions with regard to the advice they have provided:

*“I work for a school and uh, the director deals very unprofessionally with his staff… At first, I talked to him about it and eh,… and eh, afterwards also to his supervisor, and he had a conversation with him but eventually did not take any further actions… So, it really doesn’t make a difference! Eventually, you’re very limited in what you can accomplish and you have to accept that”.*

*Inertia of previous practice* was another commonly perceived attitude-related barrier. Some OPs reported to experience difficulties with changing habits and routines in order to learn new things, such as conducting a complete problem analysis and process diagnosis as described in the guideline:

*“It just takes time and energy to change something you programmed yourself to do…. and when you work under pressure, you let go of it and you resume your old routine, just to be quick and efficient”.*

#### External barriers

Three main types of external barriers were identified: worker factors, guideline factors and work-contextual barriers (Table 2).

*Worker factors* were reported as a barrier among OPs. Some OPs, for example, indicated that workers sometimes have hidden agendas aimed at a specific assessment outcome, which makes it difficult to make a correct diagnosis:

*“And then she started to accuse me of being a bad occupational physician, and how it is possible that I can decide within 15 minutes that she has to go back to work and she proposed in a very arrogant way that, maybe, it would be better for her to go wild so I could witness the nature of her illness … And then she demanded that I should contact her psychologist… and finally she ran away hysterically”.*

*Guideline factors* were perceived as barriers to using the guideline. For example, some OPs reported difficulties with having both an extensive guideline and a large background document, which makes it difficult to get a proper overview of the subject matter:

*“I think the guideline is kind of non-transparent and, of course, it’s an extensive guideline and therefore we need an extensive supporting background document as well, and the problem is, according to me, to relate all the different parts”.*

*Work-contextual factors* were also commonly perceived by the OPs as barriers and consisted of six types: work pressure/lack of time, the setting OPs operate in, organizational constraints, contracts between OHSs and employers, the policy of and collaboration with other parties and the fear of misuse of information or control by others.

*Work pressure/lack of time* was a widely perceived work-contextual barrier. OPs mentioned that a lack of time or work pressure often hindered them from following the guideline recommendations in practice:

*“If you work under time pressure and someone with psychological problems pays you a visit and you only have about 5 or 10 minutes left you might think: I shake his hand, we have a brief conversation and I just agree with him and tell him to stay home and we’ll see each other next time. This is how you may think sometimes. It’s something I don’t support at all, but it could happen for reasons of self-protection”.*

A second work-contextual barrier was the *setting OPs operate in*. Some OPs reported to experience the setting as difficult in terms of the role they have in assessments, questioning their independency towards the worker.

*Organizational constraints* were mentioned as barriers to guideline adherence. First, the policy of OHSs which affects, for instance, the work pressure of OPs, was considered as a barrier. Second, non-user-friendly (electronic health record) systems or differences in used systems among OPs were reported as a barrier to follow the guideline. OPs indicated that the systems they used and reported in were considered as non-user-friendly and made it difficult to work in accordance with the guideline. And third, lack of resources and practical constraints were experienced as barriers. OPs mentioned that working at several different locations (companies) made it very difficult to have the intervention tools readily available when needed:

*“I really need just a list and I got that kind of list, but yeah, I work at twenty different locations, resulting in that the list is lost all the time. Yes, yes I know I could keep the list in my briefcase, but there is already a lot of necessary and obligated stuff in it”.*

The fourth work-contextual barrier was *contracts between OHSs and employers*. Tight contracts in terms of the available time and reimbursement did not always match with, for example, some of the preconditions of the guideline, i.e. seeing workers within the first two weeks.

Another commonly perceived barrier was a conflicting *policy of and lack of collaboration with other parties.* First the policy of and collaboration with employers was sometimes perceived as a barrier, for instance if opinions of employers regarding how to provide care for the worker did not match with those of the OPs, or if there were no clear arrangements in terms of how to provide care. Second, the policy of and collaboration with other disciplines, such as GPs and psychologists were often reported as barriers. OPs mentioned that the policy of other disciplines, for instance psychologists, sometimes interfered with their own ideas (e.g. treatment takes too long, no attention is being paid to work):

*“Eventually he goes to see a psychologist. Well, after 8 visits I call him to ask about the situation and to make sure things are beginning to make progress. What are you doing because this doesn’t work at all? I mean, treatment right, but it’s not helping!”.*

In addition, some OPs mentioned that the collaboration with other disciplines was often suboptimal with no adequate (arrangements for) communication, reporting or feedback:

*“And I notice that some psychologists do not respond or provide feedback at all…. others do write some small comments in OCA [an electronic health record (EHR) system], but most of the time it’s like a message in the kind of: he has to take it easy or uh .. something like that”.*

Finally *fear of misuse of information or control by others* was perceived as a barrier by some of the OPs with respect to reporting in medical files, which is recommended in the guideline. OPs indicated that they sometimes feared for control of others or misuse of information and that they sometimes deliberately refrained from writing things down in patients’ medical records or write it down in such a way that it was only readable/legible for themselves:

*“You have to use abbreviations. I sometimes use those, and I know exactly what those mean but nobody else does”.*

### Solutions to overcome the barriers

In Table 3 an overview is presented of the suggested solutions the OPs came up with to address the identified barriers. They can be divided into six types of solutions.

**Table 3:** Overview of (partly) tested solutions to address barriers to using the guideline on mental health problems

1. Providing information about guideline and guideline-related tools
• Providing information about the guideline by trainer or peers
• Providing information about or referring to the availability of tools to improve guideline usage such as:
- Digital version of the guideline
- Relevant website such as www.psychischenwerk.nl (website with information and tools on psychological disorders and fatigue complaints at work)
- Relevant related guidelines and knowledge documents, such as ‘the NVAB guide for Referring’ and ‘the knowledge document STECR’ (a working guide to deal with conflicts at work).
- Relevant courses, such as the E-course MUPS (SOLK in Dutch)
- Relevant surveys, such as UBOS survey (burnout)
- Intervention tools available on G-drive of the OHS computer system
- Information letter for patients from the NHG
- Information letter for employers from the NVAB
2. Sharing experiences, tips and tricks
• Exchanging experiences in group(s) on the advantages or disadvantages of working in accordance with (certain parts of) the guideline, guideline related tools and reporting in medical files.
• Sharing tips and tricks in group, such as not accepting too tight contracts from employers, referring patients to psychiatrists with (trans)cultural expertise, tips and tricks on how to document adequately in medical files, how to use the 4DSQ (4DKL in Dutch), how to deal with suicide.
3. Presenting and discussing worker case studies
• Presenting one or more complex or successful (anonymized) worker case studies in the group and explain how they have dealt with this while other OPs provide feedback.
4. Reading and discussing peer OPs’ reporting in medical files
• Reading (anonymized) medical files of peer OPs and provide feedback.
5. Developing and adjusting tools to improve guideline usage
• Developing a format to structure the worker interview, adjusting it to individual needs and discussing ways to implement it in practice (place format on desktop, add a checklist to the format, add the format to the fan-shaped tool)
• Developing the 4DSQ tool in a digital excel version with an automatic calculation module
• Creating a book with cognitive-behavioral interventions to be used during consultation, all invented or collected by the OPs and put together in a book
• Creating a power-point presentation to educate employers or broader work-context
• Creating a referral list with healthcare providers that OPs within the group recommend
• Adjusting the fan-shaped tool with a summary of the guideline to include the format
→ Digital toolbox: creating an individual digital toolbox with a combination of above interventions as preferred by individual OPs
6. Other solutions (partly tested)
• Creating adequate (working) arrangements with respect to communication, reporting and feedback between OPs and psychologists
• Setting minimal standards for reporting for psychologists
• Initiating group conversations with worker, employer, psychologist and OP
• Organizing meetings for both psychologists and OPs to discuss the guideline on mental health problems

*NVAB* Netherlands Society of Occupational Physicians (NVAB in Dutch), *MUPS* Medically Unexplained Physical Symptoms (SOLK in Dutch), *UBOS* Utrecht Burnout Scale, *OHS* occupational health service, *NHG* Dutch College of General Practitioners (NHG in Dutch), *4DSQ* Four Dimensional Symptom Questionnaire (4DKL in Dutch), *OP(s)* occupational physician(s)

First, *providing information on the guideline and guideline-related tools* was an often tested solution to overcome barriers. Both the trainer and the OPs themselves provided knowledge to the (rest of the) OPs, such as explaining how the guideline needs to be interpreted and referring to the availability of guideline-related tools such as the digital version of the guideline, relevant websites, intervention tools and information letters for patients and employers. These solutions particularly targeted knowledge-related and attitude-related barriers.

Another often tested solution to address the identified barriers was *sharing experiences, tips and tricks* among OPs. Experiences such as the perceived value of adequate reporting in medical files and tips and tricks such as not accepting too tight contracts from employers, suggesting to refer patients to psychiatrists with (trans)cultural expertise and sharing tricks on how to document adequately in medical files. This type of solution was particularly used to address the attitude-related barriers lack of self-efficacy and inertia of previous practice and to a lesser extent external barriers.

Third, OPs suggested and tested the solution to *present one or more case studies of their workers to their peer OPs in the group* and explain how they have dealt with these particular cases, while other OPs provided feedback. This was done for both complex and successful cases and mostly targeted knowledge-related and attitude-related barriers.

Another solution the OPs came up with and tested during the year of the training was *reading and discussing each other’s reporting in (anonymized) medical files*. This solution was particularly tested to address knowledge-related barriers and attitude-related barriers such as lack of self-efficacy and inertia of previous practice.

The fifth type of tested solution was to *develop new tools or to adjust current tools to meet the needs of the individual or groups of OPs.* These tools included a format to structure the worker interview, a 4DSQ (Four Dimensional Symptom Questionnaire; 4DKL in Dutch) tool in a digital excel version with an automatic calculation module, a book with cognitive behavioral interventions with input from participating OPs from all groups to be used during consultation, a power-point presentation to educate the working-environment of workers, a referral list with names of healthcare providers of other disciplines (e.g. psychologists, psychiatrists) recommended by participating OPs from several groups, and a fan-shaped tool with a summary of the guideline. Finally, OPs created a digital toolbox consisting of a combination of the above preferred tools of individual OPs. These solutions were mainly tested to overcome knowledge-related and attitude-related barriers such as lack of self-efficacy and to a lesser extent external barriers such as practical constraints as not having intervention tools available when working at several locations.

Finally, *other solutions* were suggested by OPs, but most of them were only partly tested during the year of the training. These focused mainly on improving communication and collaboration with psychologists such as creating adequate arrangements for communication, reporting or feedback between OPs and psychologists, setting minimal standards for reporting by psychologists and organizing meetings for OPs and psychologist to discuss policy. These solutions were particularly suggested to overcome external barriers such as conflicting policy of and collaboration with other disciplines.

## Discussion

This study aimed to provide an overview of the barriers Dutch OPs perceive in adhering to the guideline on mental health problems as well as the solutions they came up with to address them. We found that a range of knowledge-related, attitude-related and external barriers hindered OPs from following the guideline on mental health problems in practice, with an emphasis on work-contextual barriers. To overcome the identified barriers, several solutions were suggested and tested during the year of training, which mostly seemed to target knowledge-related and attitude-related barriers. To optimally improve adherence to this and similar occupational mental health guidelines, it seems important to complement training and education of OPs with interventions addressing work-contextual barriers, such as changing tight contracts, or improving communication and collaboration with relevant stakeholders.

In line with Cabana’s framework [19] results from this study show that all three main types of barriers - knowledge-related, attitude-related, and external barriers - prevented OPs from following the guideline in practice. OPs lacked knowledge regarding the content of all parts of the guideline and perceived a lack of self-efficacy to perform certain recommendations and difficulties with changing habits and routines. These knowledge and attitude-related barriers may indicate that this guideline was not well introduced among the target group of OPs after being published. However, 90 % of the participants of the training had previously been educated in this guideline through short-term continuing medical education courses. Therefore, it may well be, that a multiple-session guideline training, as conducted in this study, is in fact needed to improve OPs’ knowledge, self-efficacy and skills regarding this guideline [24]. This may be necessary for process-based guidelines focusing on behavior change of the professionals, which usually cannot be accomplished in short-term courses. The current guideline asks for cognitive-behavioral skills and competencies of OPs that were not included in their professional education which predominantly focused on physical health problems. It may particularly be these types of mental guidelines for which a thorough multiple-session guideline training is useful.

External barriers and particularly work-contextual barriers were commonly reported by OPs in the training. Compared to barrier studies focusing on other types of healthcare providers (e.g. specialists [34], GPs [32, 35]), it seems that work-contextual barriers have a more prominent place among OPs, with a larger range of these barriers reported, such as work pressure/lack of time, contracts between OHSs and employers, and conflicting policy of and a lack of cooperation with employers and other disciplines. This may be related to the central role that OPs have within the (Dutch) social security system. As a consequence, they have to deal with many different stakeholders i.e. the workers, employers, the OHS, and other disciplines [36]. Changes in clinical practice and particularly worker outcomes are therefore only partly within OPs’ control; many other factors determine the outcomes. This complicates the process of implementing a guideline and asks for effective management and interventions focusing on the OPs and their larger context.

The solutions tested by OPs during the year of the training seem to preliminary focus on their widely-perceived knowledge-related and attitude-related barriers. Solutions varied from sharing information and experiences to developing or adjusting tools to facilitate the use of the guideline such as an individual digital toolbox. Solutions were only tested in practice if they were appealing to the OPs, and all were evaluated and if needed adjusted [24]. Besides from these knowledge and attitude-focused solutions, various interventions to address work-contextual barriers were suggested, such as not accepting too tight contracts in terms of available time and reimbursement, developing a referral list with high-quality healthcare providers, and developing working arrangements on communication, reporting and feedback with psychologists including minimal standards for reporting. Most of these solutions, however, were only partly tested in practice as implementing them usually required more time and the involvement of other stakeholders.

In designing guideline implementation programs it may be useful to complement guideline training and education of individuals or groups of OPs with interventions focusing on the larger context of OPs. Even if OPs are aware of the content of the guideline and have the intention to use it in practice, external barriers may still hinder them from following it in practice [19]. These include work-contextual barriers but also worker factors and guideline factors. To address work-contextual barriers, it may be useful to involve the OHSs in the implementation process. They could facilitate working conditions in terms of the available time and reimbursement needed to adequately implement the guideline in practice and provide a solution-focused environment. In addition, other stakeholders, such as employers, GPs and psychologists should be involved to align policies and to facilitate collaboration. Relevant stakeholders, including the workers themselves, should not only be engaged in the guideline implementation process, but if possible, also in the initial guideline development process or its critical revision.

A strength of this study is that the perceived barriers were assessed within the same group(s) of OPs over a longer period of time. Whereas many barrier studies have been conducted in other healthcare settings e.g. [32, 34, 35, 37], most studies have only measured barriers cross-sectionally. Perceived barriers, however, may vary across different stages of implementation [20]. For instance, knowledge-related barriers may be most relevant at the beginning of the implementation process, whereas, once these have been removed, the existence of attitude-related and external barriers may come forward [24]. Assessing barriers over a period of time, therefore provides a more complete picture and gives room to all types of barriers. Second, the solutions OPs came up with were tailored to these barriers, taking into account the context of daily practice, and if needed adjusted during the year of the training [24]. In addition, whereas the overview of proposed solutions may not be sufficient to target all barriers, it consists of solutions that were all suggested and appreciated by the target group itself, which is crucial for successful implementation and behavior change [22]. Whether practicing these solutions positively affects guideline adherence needs to be further examined.

Some limitations should also be considered in interpreting our findings. First, whereas the barriers were assessed over a longer period of time, the qualitative design of this study (and the fact that in each session a new topic of the guideline was discussed), did not allow us to analyze the results longitudinally. Rather, we provided an overview of all barriers that were perceived by participating OPs during the year of the training. Future quantitative studies could focus on the evolution of barriers in relation to the proposed solutions and the different stages of implementation of the target group(s). In addition, results of this study are based on a Dutch occupational guideline on mental health, which limits the generalizability of our findings. The few occupational mental health guidelines that are available worldwide have comparable content, yet varying levels of reporting quality [10]. Also, the Dutch context differs from that of others countries [27, 36], which may affect the perceived barriers and related solutions to some extent. Nevertheless, we believe the overviews of barriers and solutions is a valuable basis to be used in developing and implementing similar guidelines in other countries.

## Conclusions

Despite their major impact on sickness absence, only few clinical occupational health guidelines on mental health problems are available worldwide [10] and, thus far, little is known on how to successfully implement these guidelines in practice. Results from this study suggest that an extensive guideline training and education for groups of OPs, to target their knowledge and attitude-related barriers in adhering to the guideline, may indeed be useful. To optimally implement this or similar guidelines, however, it seems necessary to address work-contextual barriers and other external barriers as well, by focusing on the larger context of OPs. Engaging all relevant stakeholders (e.g. workers, employers, OHSs, other disciplines) in the guideline implementation process, as well as in its initial development process or revision is strongly recommended.

## Abbreviations

OP(s), occupational physician(s); OHS(s), occupational health service(s); NVAB, Netherlands Society of Occupational Physicians (NVAB in Dutch); RTW, return-to-work; GP, general practitioner; PDCA, plan-do-check-act; UWV, Dutch Institute for Employee Benefit Schemes (UWV in Dutch); EHRS, electronic health record system; MOPS, Medically Unexplained Physical Symptoms (SOLK in Dutch); UBOS, Utrecht burnout scale; NHG, Dutch College of General Practitioners (NHG in Dutch); 4DSQ, Four Dimensional Symptom Questionnaire (4DKL in Dutch)
